# Stereotactic Accelerated Partial Breast Irradiation for Early-Stage Breast Cancer: Rationale, Feasibility, and Early Experience Using the CyberKnife Radiosurgery Delivery Platform

**DOI:** 10.3389/fonc.2016.00129

**Published:** 2016-05-23

**Authors:** Olusola Obayomi-Davies, Thomas P. Kole, Bridget Oppong, Sonali Rudra, Erini V. Makariou, Lloyd D. Campbell, Hozaifa M. Anjum, Sean P. Collins, Keith Unger, Shawna Willey, Eleni Tousimis, Brian T. Collins

**Affiliations:** ^1^Department of Radiation Medicine, Lombardi Comprehensive Cancer Center, Georgetown University Hospital, Washington, DC, USA; ^2^Betty Lou Ourisman Breast Health Center, Lombardi Comprehensive Cancer Center, Georgetown University Hospital, Washington, DC, USA; ^3^Department of Radiology, MedStar Georgetown University Hospital, Washington, DC, USA

**Keywords:** breast cancer, breast SBRT, CyberKnife breast, partial breast irradiation, APBI, stereotactic radiosurgery, breast radiosurgery

## Abstract

**Purpose:**

The efficacy of accelerated partial breast irradiation (APBI) utilizing brachytherapy or conventional external beam radiation has been studied in early-stage breast cancer treated with breast-conserving surgery. Data regarding stereotactic treatment approaches are emerging. The CyberKnife linear accelerator enables excellent dose conformality to target structures while adjusting for target and patient motion. We report our institutional experience on the technical feasibility and rationale for stereotactic accelerated partial breast irradiation (SAPBI) delivery using the CyberKnife radiosurgery system.

**Methods:**

Ten patients completed CyberKnife SAPBI (CK-SAPBI) in 2013 at Georgetown University Hospital. Four gold fiducials were implanted around the lumpectomy cavity prior to treatment under ultrasound guidance. The synchrony system tracked intrafraction motion of the fiducials. The clinical target volume was defined on contrast enhanced CT scans using surgical clips and post-operative changes. A 5 mm expansion was added to create the planning treatment volume (PTV). A total dose of 30 Gy was delivered to the PTV in five consecutive fractions. Target and critical structure doses were assessed as per the National Surgical Adjuvant Breast and Bowel Project B-39 study.

**Results:**

At least three fiducials were tracked in 100% of cases. The Mean treated PTV was 70 cm^3^ and the mean prescription isodose line was 80%. Mean dose to target volumes and constraints are as follows: 100% of the PTV received the prescription dose (PTV30). The volume of the ipsilateral breast receiving 30 Gy (V30) and above 15 Gy (V > 15) was 14 and 31%, respectively. The ipsilateral lung volume receiving 9 Gy (V9) was 3%, and the contralateral lung volume receiving 1.5 Gy (V1.5) was 8%. For left-sided breast cancers, the volume of heart receiving 1.5 Gy (V1.5) was 31%. Maximum skin dose was 36 Gy. At a median follow-up of 1.3 years, all patients have experienced excellent/good breast cosmesis outcomes, and no breast events have been recorded.

**Conclusion:**

CyberKnife stereotactic accelerated partial breast irradiation is an appealing technique for partial breast irradiation offering improvements over existing APBI techniques. Our early findings indicate that CK-SAPBI delivered in five daily fractions is feasible, well tolerated, and is a reliable platform for delivering APBI.

## Introduction

Breast-conserving therapy (BCT) is the preferred treatment approach for early-stage breast cancer and numerous randomized controlled studies have demonstrated equivalent overall survival for patients receiving breast-conserving surgery with whole breast irradiation (WBI) compared with patients treated by mastectomy alone (1–4). These studies demonstrate ~70% reduction in local recurrence with the addition of adjuvant radiation after breast-conserving surgery (5) and a reduction in the risk of breast cancer death by one-sixth at 15 years (6). Despite the known advantages of BCT, its utilization remains low with only 10–80% of eligible patients completing treatment. Prolonged treatment time, cost, distance to treatment facilities, and patient inconvenience have been implicated as possible deterrents to BCT (7–10).

Accelerated partial breast irradiation (APBI) delivers a high dose of radiation therapy to the region around the lumpectomy cavity (11, 12). However, current APBI techniques have significant drawbacks. For instance, balloon-based brachytherapy applicators may not be suitable for cavities close to the skin surface or for irregularly shaped cavities to which the balloon cannot conform (13). Air and fluid pockets in the lumpectomy cavity can create dosimetric aberrations requiring adjustments (14). External beam APBI (EB-APBI) is subject to intra-fractional motion, surface deformation, and treatment set-up uncertainties that have to be accounted for with increases in the planning target volume (PTV) resulting in larger amounts of normal breast tissue receive high-dose irradiation and increased risk of poor cosmesis (15–17).

CyberKnife stereotactic accelerated partial breast irradiation (CK-SAPBI) offers technical improvements in partial breast irradiation using real-time tracking, respiratory motion management, and submillimeter accuracy with few technical limitations (18, 19). Reduced target and treatment uncertainty allows for treatment intensification, maximal target coverage reminiscent of high-dose brachytherapy while protecting normal breast tissue from unnecessary high-dose irradiation (20–22). With these technical improvements, we expect more women will be eligible for CK-SAPBI with similar local control, increased patient convenience, and improved cosmesis compared to existing EB-APBI techniques. We present our early institutional experience with the technical set-up, treatment planning, and dosimetric parameters of 10 patients receiving CK-SAPBI.

## Materials and Methods

### Patient Selection

This restrospective analysis was approved by the hospital institutional review board. Most patients were “suitable” or “cautionary” according to ASTRO consensus guidelines for APBI (23). Patients were aged ≥48 years with stage 0 or I histologically confirmed invasive non-lobular carcinoma or ductal carcinoma *in situ* (DCIS). Tumor size was required to be ≤2 cm in maximum diameter and surgically excised with negative margins ≥2 mm. Patients with invasive ductal carcinoma underwent negative sentinel sampling.

Prior to CK-SAPBI treatment, all patients were evaluated by a single board certified radiation oncologist and were seeking adjuvant treatment with PBI only. Patients with large seromas or hyper deformable breast tissue (poor breast integrity) were not offered CK-SAPBI. These patients were deemed to be poor candidates for fiducial tracking purposes based on previous unpublished institutional experience. All patients opted for CK-SAPBI after discussing risks and alternatives, including standard WBI. Written informed consent was obtained outlining the above discussion.

### Treatment Planning and Immobilization

Prior to treatment, four 2-mm gold fiducials (Best Medical International Inc.) were implanted around the lumpectomy site under ultrasound guidance by a single board certified radiologist. Pre-surgical imaging was used to guide optimal fiducial placement. For optimal tracking, fiducials must be non-coplanar and have an angular separation of at least 15° between any two fiducials. Thus, fiducials were placed at the 12:00, 6:00, 10:00, and 4:00 rad relative to the lumpectomy cavity to satisfy these constraints. Contrast-enhanced 1-mm CT scans were obtained in the supine position with patient arms placed by their sides approximately 1 week after fiducials were placed. No breast immobilization devices were required.

The CT images were exported to the MultiPlan treatment planning software (Accuray Incorporated, Sunnyvale, CA, USA). The lumpectomy cavity and post-surgical changes were identified and a target region was manually defined around them with additional soft tissue margins confined to the breast tissue and skin contour to generate a clinical target volume (CTV). Pre-operative imaging was used to assist target definition when artifacts or tissue density interfered with delineation. A uniform 5-mm expansion was used to generate the PTV. The arms were contoured and no beam entry or exit was permitted through the arms. The ipsilateral breast, contralateral breast, skin, chest wall, lungs, heart, and thyroid were delineated and designated avoidance structures.

Inverse CyberKnife plans were generated to deliver 30 Gy in five fractions to the PTV over consecutive days. This corresponds to a biologically effective dose equivalent to 50 Gy in 25 fractions assuming an α/β for tumor control of 4 Gy (24). Formenti initially published their experience using this fractionation scheme with excellent local control rates and good cosmesis at a median follow-up of 5 years (25). This dose scheme was also employed in the randomized controlled phase III trial of WBI versus PBI using IMRT (26).

All patient plans were generated using the MultiPlan treatment planning system (version 4.6.0). Dose distribution calculations were performed using Monte Carlo dose calculation algorithm with heterogeneity correction. Dose volume histogram (DVH) assessments were performed to determine if institutional dose constraints were met. For the purpose of this study, DVH analyses were performed and compared to external beam PBI constraints in the National Surgical Adjuvant Breast and Bowel Project B-39/Radiation Therapy Oncology group 0413 (NSABP/RTOG) study.

### Follow-up

Follow-up visits were conducted by the treating physician 4 weeks after the completion of treatment and at scheduled intervals thereafter per routine practice. Breast examinations and scheduled mammograms were performed to assess local control and changes in the appearance of the breast. Toxicity and cosmesis were assessed by a single physician using the Harvard scale of cosmetic outcome.

## Results

**Figure 1:**
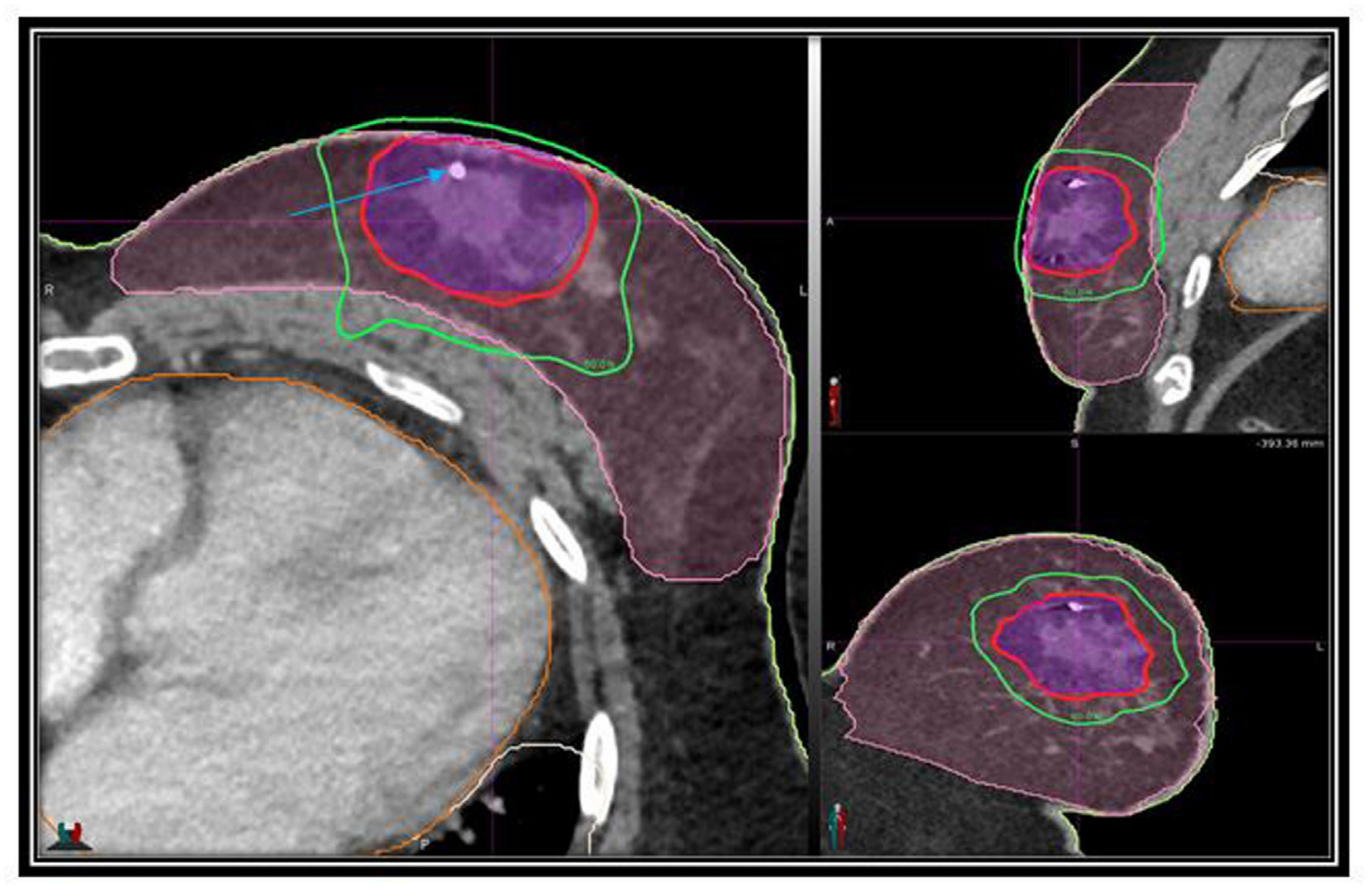
**Axial, sagittal, and coronal views of the treatment planning CT scan demonstrating the PTV (purple) and normal breast (pink), isodose lines shown as follows: 100% of the prescription dose, red line; 50% of the prescription dose, green line; arrow points to gold fiducial marker**.

**Figure 2:**
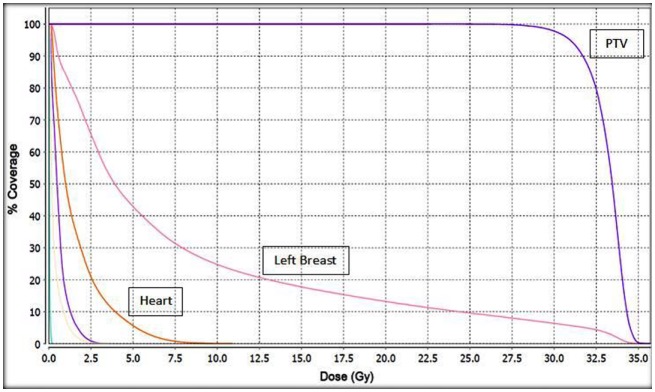
**Cumulative dose volume histogram (DVH) for the target PTV and normal tissues**. This plan was normalized to deliver 30 Gy to the PTV. Unlabeled structures: left lung; purple. Right lung; yellow. Right breast; light blue.

Between 2/2013 and 12/2013, 10 patients received CK-SAPBI. Table 1 provides a summary of patient demographics and tumor characteristics. All four fiducials were successfully tracked during treatment in 60% of cases. At least three fiducials were successfully tracked during treatment in all patients. The mean number of beams delivered was 155 (119–194). Figures 1 and 2 illustrate a typical treatment plan and DVH. The mean treated PTV volume was 70 cm^3^ (range = 35–142 cm^3^) with a mean prescription isodose line of 80% (range = 77–87%).

**Table 1:** **Patients and tumor characteristics**.

Value
Age (years)	
Mean (range)	61 (48–77)
Tumor histology	
IDC	3
DCIS	7
Stage	
Tis	7
T1b	2
T1c	1
Tumor size (centimeter)	
Mean (range)	0.95 (0.3–2.0)
IDC	1.2 (1.0–1.5)
DCIS	0.8 (0.3–2.0)
Laterality	
Right	6
Left	4
Quadrant	
Central	1
UOQ	5
UIQ	3
LIQ	1
Nodal stage	
NX	7
N0	3
ASTRO APBI Consensus Group	Suitable-3 Cautionary-6 Unsuitable-1
Other	
ER positive	9
Cup size	B-2, C-4, D-1, DD-1

*IDC, invasive ductal carcinoma; DCIS, ductal carcinoma in situ; UOQ, upper outer quadrant; UIQ, upper inner quadrant, LIQ, lower inner quadrant; NX, lymph nodes not sampled; N0, sampled lymph nodes were negative; ER, estrogen receptor*.

Dose volume histogram analysis of organs at risk is summarized in Table 2. In four patients, the tumor beds were located in the inner quadrant increasing the contralateral breast dose. In each case, less than 10% of the contralateral breast volume received above 2 Gy for the entire treatment. One patient had an ipsilateral lung V9Gy above 15%. This patient had a deep-seated tumor close to the chest wall and was an outlier in our series.

**Table 2:** **Dose limitations for normal tissue based on the NSABP/RTOG B-39 protocol for patients treated with CyberKnife SAPBI to a total dose of 30 Gy delivered in five fractions (*n* = 10)**.

**Structure**	**Constraint**	**CyberKnife Treatment (mean, range)**
Ipsilateral breast	V30 < 35%	14%, 3–26%
	V15 < 60%	31%, 8–58%
Contralateral breast	D_max_ < 1 Gy	3 Gy, 0–11 Gy
Ipsilateral lung	V9 < 15%	3%, 0–17%
Contralateral lung	V1.5 < 15%	8%, 0–21%
Heart (left breast)	V1.5 < 40%	31%, 7–43%
Heart (right breast)	V1.5 < 5%	18%, 0–37%
Thyroid	D_max_ < 1 Gy	<1 Gy, 0–1.4 Gy
Skin	D_max_ < 36 Gy	32 Gy, 28–36 Gy
Chest wall	D_max_ < 36 Gy	26 Gy, 13–33 Gy

*SAPBI, stereotactic accelerated partial breast irradiation; NSABP, National Surgical Adjuvant Breast and Bowel Project; RTOG, Radiation Therapy Oncology Group; 3D-CRT, three-dimensional conformal radiotherapy*.

**Figure 3:**
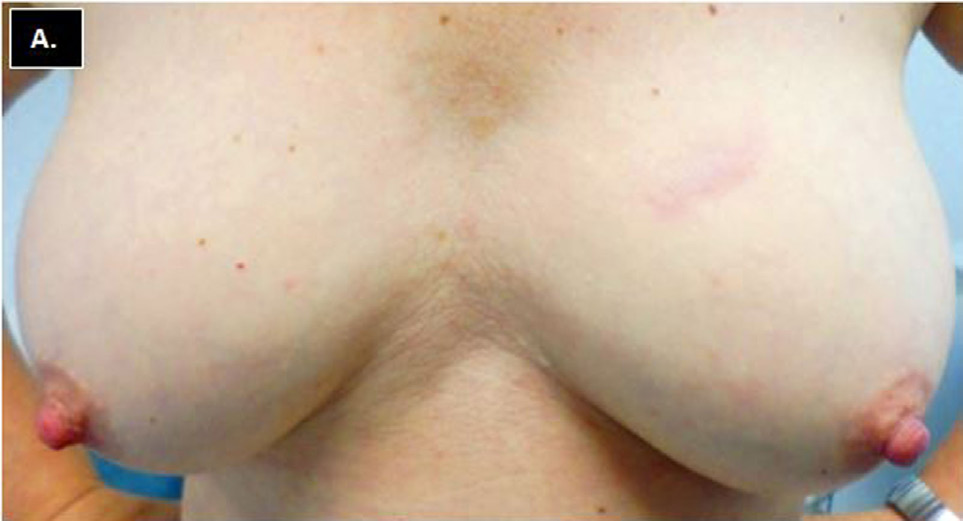
**Frontal view of bilateral breasts showing excellent breast cosmesis 18 months following CyberKnife SAPBI**. *SAPBI, Stereotactic accelerated partial breast irradiation*.

Minimal toxicity was observed following treatment. One patient has not returned for follow-up. Among returning patients, with a maximum follow-up of 1.6 years (range = 0.1–1.5 years, median 1.25 years), no adverse breast events have been recorded. One patient had grade 1 skin induration 3 weeks after treatment which resolved without intervention. No other skin, lung, chest wall, or breast toxicity has been observed. All patients experienced excellent/good cosmetic outcomes following treatment. Figure 3 illustrates a patient with a left-sided upper inner quadrant CK-SAPBI treatment with excellent cosmetic outcome 15 months following treatment.

## Discussion

Partial breast irradiation is a promising treatment technique for the treatment of early-stage breast cancer. Multiple randomized trials are evaluating treatment outcomes but early available data are encouraging and suggest low recurrence rates in well-selected women. CK-SAPBI is a convenient alternative with meaningful technical improvements compared to existing partial breast irradiation techniques.

The CyberKnife radiosurgery system has been used successfully at our institution since early 2002. We sought to apply our vast experience in treating cranial, spine, lung, prostate, and pancreatic tumors to adjuvant APBI. We carefully selected patients with favorable pathologic features to minimize the risk of ipsilateral breast recurrence. Furthermore, we avoided patients with large seromas and poor breast integrity to avoid sub-optimal fiducial tracking. Our CK-SAPBI experience thus far is encouraging and confirms our presumptions about the feasibility of the CyberKnife radiosurgery system in delivering targeted APBI. The minimal treatment volumes and high dose conformity are reminiscent of high dose rate interstitial brachytherapy without its known technical challenges and invasive requirements making CK-SAPBI quite appealing.

CyberKnife stereotactic accelerated partial breast irradiation offers additional advantages of fewer treatments delivered and increased patient comfort due to lack of a second surgical procedure to place the brachytherapy applicator. Furthermore, concerns regarding worse cosmetic outcomes with APBI are likely abated with CK-SAPBI. Prior to publication of the RAPID trial interim analysis showing unacceptable worse cosmesis in the 3D-CRT arm, lager treatment volumes had been associated with worse breast cosmesis after EB-PBI (27). With a mean treated PTV of 70cm^3^ in our series, we expect favorable long-term cosmesis and at median follow-up of 1.25 years cosmetic outcomes remain excellent/good.

While the technical advantages over existing PBI platforms are evident, potential disadvantages exist with CK-SAPBI. First, since fiducials are required to track target motion, proper fiducial positioning is crucial. Fiducial migration can occur after placement, hindering optimal real-time tracking. As such, our practice is to obtain our treatment planning imaging at least 7 days following fiducial implantation to allow for tissue recovery. Moreover, fiducial tracking can be sub-optimal in patients with poor breast integrity and large post-operative seromas. Second, CK-SAPBI treatment times are prolonged compared to 3D-CRT and IMRT treatments. However, this trade-off is likely acceptable given the reduced treatment volume and higher treatment conformity afforded by CyberKnife treatment delivery. Finally, delineation of the lumpectomy cavity can be difficult. This difficulty is particularly pronounced with oncoplastic reconstructions and when clips have not been placed to delineate the surgical cavity. A commercially available three-dimensional (3D) bioabsorbable tissue marker (BioZorb, Focal Therapeutics, Portola Valley, CA, USA) offers profound improvements in this regard. The rigid lattice of the BioZorb preserves the 3D location of the lumpectomy cavity even after oncoplastic reconstruction (28).

Our study is limited by its small size, short follow-up, and retrospective nature. The results are nonetheless encouraging and we have concluded that CK-SAPBI is a reasonable non-invasive APBI delivery platform in early-stage breast cancer. Additional patients will be evaluated to determine the optimal patient population for this treatment approach. To this effect, we have commissioned a prospective phase II trial to further evaluate CK-SAPBI in early-stage breast cancer.

## Conclusion

Stereotactic accelerated partial breast irradiation via CyberKnife is a suitable platform for partial breast irradiation offering improvements over existing APBI techniques. Our experience confirms previous reports regarding CK-SAPBI and suggests that this technique is feasible for the delivery of APBI.

## Author’s Note

Meeting Presentation: Preliminary results of this study were presented at the 97th annual American Radium Society meeting in 2015 in Kauai, HI from May 2nd to 5th, 2015.

## Author Contributions

OO-D, BC, TK, LC, BO, SW, ET, EM, and SR all contributed to study concept, design, and/or acquisition of data. OO-D, TK, HA, KU, and BC completed the data collection. BC and OO-D contributed to the data analysis. OO-D was responsible for drafting the manuscript. All authors contributed to revising and giving final approval to the manuscript. All authors agree to be accountable for all aspects of the work including its accuracy and integrity.

## Conflict of Interest Statement

BC, SC, and OO-D are paid speakers for Accuray. The remaining authors have no competing interests.
